# The Effect of Mobile App Home Monitoring on Number of In-Person Visits Following Ambulatory Surgery: Protocol for a Randomized Controlled Trial

**DOI:** 10.2196/resprot.4352

**Published:** 2015-06-03

**Authors:** Kathleen A Armstrong, Peter C Coyte, R Sacha Bhatia, John L Semple

**Affiliations:** ^1^ Division of Plastic & Reconstructive Surgery Department of Surgery University of Toronto Toronto, ON Canada; ^2^ Institute of Health Policy, Management and Evaluation University of Toronto Toronto, ON Canada; ^3^ Women’s College Hospital Institute for Health System Solutions and Virtual Care Women's College Hospital Toronto, ON Canada; ^4^ Department of Ambulatory Surgery Women's College Hospital Toronto, ON Canada

**Keywords:** mobile apps, randomized controlled trial, cost-effectiveness, ambulatory surgical procedures, ambulatory monitoring, technology assessment

## Abstract

**Background:**

Women’s College Hospital, Toronto, Canada, offers specialized ambulatory surgical procedures. Patients often travel great distances to undergo surgery. Most patients receiving ambulatory surgery have a low rate of postoperative events necessitating clinic visits. However, regular follow-up is still considered important in the early postoperative phase. Increasingly, telemedicine is used to overcome the distance patients must travel to receive specialized care. Telemedicine data suggest that mobile monitoring and follow-up care is valued by patients and can reduce costs to society. Women’s College Hospital has used a mobile app (QoC Health Inc) to complement in-person postoperative follow-up care for breast reconstruction patients. Preliminary studies suggest that mobile app follow-up care is feasible, can avert in-person follow-up care, and is cost-effective from a societal and health care system perspective.

**Objective:**

We hope to expand the use of mobile app follow-up care through its formal assessment in a randomized controlled trial. In postoperative ambulatory surgery patients at Women’s College Hospital (WCH), can we avert in-person follow-up care through the use of mobile app follow-up care compared to conventional, in-person follow-up care in the first 30 days after surgery.

**Methods:**

This will be a pragmatic, single-center, open, controlled, 2-arm parallel-group superiority randomized trial comparing mobile app and in-person follow-up care over the first month following surgery. The patient population will comprise all postoperative ambulatory surgery patients at WCH undergoing breast reconstruction. The intervention consists of a postoperative mobile app follow-up care using the quality of recovery-9 (QoR9) and a pain visual analog scale (VAS), surgery-specific questions, and surgical site photos submitted daily for the first 2 weeks and weekly for the following 2 weeks. The primary outcome is the total number of physician visits related to the surgery over the first 30-days postoperative. The secondary outcomes include (1) the total number of phone calls and emails to a health care professional related to surgery, (2) complication rate, (3) societal and health care system costs, and (4) patient satisfaction over the first 30 days postoperative. Permutated-block randomization will be conducted by blocks of 4-6 using the program ralloc in Stata. This is an open study due to the nature of the intervention.

**Results:**

A sample of 72 (36 patients per group) will provide an E-test for count data with a power of 95% (*P*=.05) to detect a difference of 1 visit between groups, assuming a 10% drop out rate. Count variables will be analyzed using Poisson regression. Categorical variables will be tested using a chi-square test. Cost-effectiveness will be analyzed using net benefit regression. Outcomes will be assessed over the first 30 days following surgery.

**Conclusions:**

We hope to show that the use of a mobile app in follow-up care minimizes the need for in-person visits for postoperative patients.

**Trial Registration:**

Clinicaltrials.gov NCT02318953; https://clinicaltrials.gov/ct2/show/NCT02318953 (Archived by WebCite at http://www.webcitation.org/6Yifzdjph).

## Introduction

### Background and Rationale

Women’s College Hospital (WCH) in Toronto offers specialized surgical procedures, including breast reconstruction following mastectomy for breast cancer. The goal of surgery is to restore a breast mound and improve quality of life in survivors [1-3]. Patients often travel significant distances after choosing to undergo surgery at WCH. Increasingly, telemedicine is used to overcome the distance patients must travel to receive specialized care. Telemedicine is the use of medical information exchanged from one site to another via electronic communication to improve patients’ health status [4].

Several countries with large rural populations have utilized telemedicine services to improve access to care. In India, telemedicine is used to follow postoperative patients with parathyroid disease and thyroid disease. Many of these patients live more than 1000 kilometers away from the tertiary hospital [4]. In Ecuador, a mobile surgical program utilizes telemedicine to provide routine preoperative and postoperative care and demonstrated improved resource utilization through elimination of redundant examinations and superfluous travel [5]. These studies demonstrate that it is possible to improve access to necessary services with cost- and time-savings to patients and providers.

Currently, Women’s College Hospital is using a mobile app (QoC Health Inc, Toronto) to replace in-person follow-up care after surgery for breast reconstruction patients. A feasibility pilot study evaluating the quality of recovery at home using this mobile device has been completed. Preliminary findings suggest that mobile follow-up care adequately detects postoperative complications and is potentially cost effective from a health system perspective [6,7].

Breast reconstruction is an underused option, despite its well-known psychosocial benefits in breast cancer patients [2,8]. Recent studies suggest that low income and nonurban residence are associated with decreased rates of breast reconstruction [9]. Telemedicine data suggest it can join patients and providers separated by physical distance at a reduced cost to society [10-12]. By reducing costs to society, of which a component represents costs borne by patients, we hope to improve access to breast reconstruction among breast cancer survivors.

### Knowledge to Date

#### Why Is Telemedicine an Important Initiative in Ontario?

The Ontario government has devised an action plan to change health care delivery so that it meets the fiscal challenges and needs of an aging population [13]. The Ontario Action Plan identifies technology as an opportunity to reduce the growth in health care costs and eliminate the barrier of distance. The action plan states that Ontarians should have access to the right care, at the right time, in the right place. This project speaks directly to these objectives by providing breast reconstruction patients with timely contact (the right time) with their surgeon (the right care) from the comfort of their home (the right place) [13]. This technology has the potential to address wait times by freeing up specialty surgeon clinic time to see new consults and non-urgent visits to the emergency department by providing direct surgeon contact.

#### Why Is an Elective Ambulatory Population Suitable for Telemedicine Follow-Up?

In general, morbidity and mortality following ambulatory surgery is exceedingly low [14]. This is more apparent in an ambulatory facility where various patient selection rules result in the treatment of largely American Society for Anesthesia (ASA) class I and II patients [14]. These patients are considered healthy or with mild systemic disease, respectively. Complication rates in this subset of breast reconstruction patients are approximately 5% [15]. Low complication rates mean that postoperative intervention is exceedingly unlikely. Previous studies have found that after a tonsillectomy or adenoidectomy, telephone follow-up care with standardized questionnaires is as safe as standard follow-up care and offers considerable cost reduction and patient convenience [16]. Similar telephone follow-up has also been used successfully in elective open hernia repairs and laparoscopic cholecystectomy [17]. Others have shown that planned outpatient appointments after uncomplicated surgery are neither necessary nor cost effective [18]. A “no planned follow-up” saves money for hospitals and patients. However, postoperative follow-up is valued by patients and important for the continuity of care [18]. In this way, telemedicine follow-up care offers a middle ground between conventional in-person follow-up care and no planned follow-up care.

#### Why Use the QoC Health Inc Mobile App?

The QoC Health Inc mobile app allows patients to submit photos and answers to a validated quality of recovery (QoR) questionnaire and Visual Analog Scale (VAS) for the first 30 days postoperative using their mobile device [19]. A feasibility study, including 30 breast reconstruction patients, showed once daily reporting was well tolerated even in the first week postoperative period. There were high levels of satisfaction with the mobile device and app (3.9/5) and low levels of anxiety in knowing that their surgeon was monitoring their recovery [6]. One wound infection and one early dehiscence was picked up using the mobile phone app, leading to immediate intervention. Surgeons were able to follow patient reports on a Web portal. Post-pilot surveys reported an overall positive experience. All patients attended the standard in-person follow-up care visit at 1 and 4 weeks postoperative. Surgeons felt that at least one early follow-up clinic visit could be eliminated when using the remote monitoring technology. This was an unexpected finding [6].

The following proposed catalyst study builds on this pre-existing data by actually eliminating one or more in-person postoperative follow-ups and performs a cost-effectiveness assessment. Demonstrating cost-effectiveness in an elective ambulatory postoperative population would promote its use in similar surgical settings.

### Research Question

In postoperative ambulatory breast reconstruction patients at WCH, can we avert in-person follow-up care through the use of a mobile app compared to conventional, in-person follow-up care in the first 30 days following surgery?

## Methods

### Design Overview

This will be a pragmatic, single-center, open, controlled, 2-arm parallel-group superiority randomized trial comparing mobile app and in-person follow-up care over the first 30 days following surgery. Permutated-block randomization will be conducted by blocks of 4-6 using the program ralloc in Stata statistical software [20].

### Study Setting

All study participants will be recruited from WCH. This has been an exclusively ambulatory hospital since September 2011, meaning that all surgical patients go home the same day or the next day after surgery. The Chief of Surgery (JS) at WCH was instrumental in developing this mobile app and is a champion of eHealth interventions. Due to physician interest, the app will be adapted for use in other surgical patient populations, including arthroscopic orthopedic, thyroid, and parathyroid surgical patients. WCH has the infrastructure to support research and to participate in knowledge dissemination and uptake activities.

### Characteristics of Ambulatory Breast Reconstruction Patients

This patient population is female between 18-70 years of age. Due to the limited availability of breast reconstruction in Ontario [9], patients travel from all over Ontario to receive breast reconstruction. The types of breast reconstruction performed at WCH include immediate (ie, at time of mastectomy) or delayed reconstruction using one-stage reconstruction with alloderm and implants or two-stage reconstruction using expanders that are later exchanged for implants, immediate or delayed reconstruction using a pedicled transverse rectus abdominis myocutaneous flap or pedicled lattisimus dorsi flap, bilateral breast reduction, and fat grafting.

Common indications for breast reconstruction include breast cancer surgery, prophylactic mastectomy due to familial risk factors, and breast hypertrophy qualifying for breast reduction under OHIP (Ontario Health Insurance Plan).

All patients are within the ASA class I, II, and III representing patients with good health, mild systemic disease, and severe systemic disease, respectively. The majority of patients fall within ASA classes I and II. All patients are nonsmokers with a body mass index (BMI) ≤30. Approximately 50% of breast cancer patients receive pre-operative radiation with or without chemotherapy. These factors affect complication rates and therefore determine if the groups are balanced after randomization.

### Selection Criteria

The inclusion criteria are patients undergoing breast reconstruction at WCH. They must be able to use a mobile device and communicate in English. The exclusion criterion are (1) patients who are smokers, because smokers carry increased rates of complication and both surgeons have a policy to solely operate on non-smokers (minimum smoke-free period of 1 month leading to surgery). Patients must not (2) suffer from chronic pain, (3) be taking narcotic (morphine-like) medication for pain on a regular basis, and (4) have an allergy to local anesthetics or morphine-like medications. Pain ratings captured in the VAS and QoR-9 are important for judging quality of postoperative recovery. Pre-existing pain or an inability to take narcotics would compromise the reliability of these measures.

Patients with hearing or speaking impairments will be accommodated with the help of translators. The person who regularly attends visits with the patient will facilitate this, or if no such person is available, we will use a hospital translator. All patients will receive an explanation of the study and the consent form in writing. All material will be understandable by patients with a grade 6 reading level. If our patients have lower than a grade 6 reading level, we will ask them if there is a family member at home who could assist them with the use of the mobile device.

### Intervention

Eligible patients will be randomized in equal proportions (1:1) between mobile app and in-person follow-up care. All patients will receive what is currently the medical standard of care in this hospital. Patients in the conventional follow-up group will have a planned clinic follow-up at 4 weeks postoperative. This is the follow-up schedule currently used by both surgeons. At these scheduled follow-ups, patients will be asked to complete the VAS to assess pain and the QoR-9.

The mobile app follow-up group will have no planned in-person follow-up at 1 week and 4 weeks postoperative. However, these visits will be replaced with surgical site examination via submitted photos, VAS, and QoR-9 questionnaire monitoring. All of this information is submitted via the mobile app (QoC Health Inc, Toronto). Patient reporting will begin following discharge from the recovery room. Since 75% of complications occur within the first 2 weeks of discharge [21], we will use daily monitoring for 2 weeks and then weekly monitoring for 4 weeks. The data entered through the mobile phone app will reach a double-encrypted server. The surgeon will then use a wireless interface to access that data and monitor the patient’s condition (not in real time). High pain scores will be flagged in the database for quick viewing. Any red flags will prompt in-person follow-up. Physicians will summarize the clinical findings recorded by the mobile app at 1 week and 4 weeks postoperative using the prototypical subjective, objective, assessment, and plan (SOAP) note.

### Primary Outcome

The total number of physician visits (including specialist, family physician, and emergency department) related to the surgery. These data will be captured at 4 weeks after surgery.

### Secondary Outcomes

The total number of health care telephone calls and emails (including specialist, family physician, and emergency department) related to the surgery will be captured at 4 weeks after surgery.

We will also record and report all complications occurring within the 30-day period. This was chosen based on literature surrounding postoperative complications in the first 30 days [21]. This will be captured at 4 weeks after surgery. The complication rate within this patient population is 4%, with 1% rate of reoperation. The most common complications are superficial skin infections managed with a short course of oral antibiotics. In the pilot study, all (1/30) superficial skin infections were picked up by the mobile phone app and antibiotic prescriptions were called in to the patient’s local pharmacy [6]. Rare non-serious complications include seromas and wound dehiscence. All wound dehiscence (1/30) were picked up by the app in the pilot study. These both warrant a trial of conservative (watch-and-wait) therapy. After failed conservative therapy, seroma may be drained via ultrasound guided needle aspiration. Wound dehiscence may require surgical management under local anesthetic in the clinic or, if more involved, under regional anesthetic in the operating room. Rare and potentially serious complications include hematomas. If a hematoma is small and non-expanding, it can be managed conservatively (watch-and-wait). If it is larger and expanding, it may require urgent (<24 hours) evacuation in the operating room. This type of urgent situation presents to the emergency department, not to a clinic visit.

There is no potential for clinical compromise in the telemedicine group. If anything, superficial skin infections may be identified and treated earlier. Any study participant can schedule a face-to-face postoperative visit with the surgeon at any time; however it is anticipated that the mobile phone follow-up care will eliminate the need for one or more clinic visits. Devices may be returned to the clinic in-person or by standard mail.

A societal perspective will be adopted wherein all costs are assessed irrespective of the payer [22]. This perspective was chosen based on the US Panel on Cost-Effectiveness in Health and Medicine recommendations. This recommendation is meant to improve comparability and consistency across studies [23]. Furthermore, while a broad societal perspective will be adopted, results will also be presented using a narrower health system perspective that may be of key interest to health administrators and policy decision makers. This alternative perspective focuses on costs borne within the health system and excludes external costs as well as costs borne by patients and their caregivers.

Currently, there are no validated questionnaires that capture patient satisfaction with postoperative care. We have created a post-pilot survey that captures patient satisfaction with the care and information received. All answers are recorded using a 5-point Likert scale (Multimedia Appendix 1). We will also use the QoR-9 scores and VAS recorded at 4 weeks postoperative. Psychometric properties of the QoR-9 include convergent validity and discriminant construct validity. There is also good interrater agreement and internal consistency [24]. The test-retest reliability was 0.61 (*P*<.001). The preferred cut-off is 0.7; however, the QoR-9 was still favored over the QoR-40 due to its ease of use (<2 minutes required to complete the survey) [24].

### Sample Size

The average breast reconstruction patient attends two in-person follow-up visits within the first month after surgery. If we assume that we can avert at least one in-person visit in the mobile app arm and that we have equal number of participants in both groups, we can use the E-test for count data to generate a sample size of 64 (32 patients per group) at a power of 95% (*P*=.05). The E-test is more robust and more powerful than the Conditional test (C-test) originally described to compare two counts. If we assume a 10% dropout rate, we will increase our sample size target to 36 patients per group.

### Informed Consent

The Research Ethics Board (REB) at WCH and the University of Toronto will review the project proposal. Amendments will be made to appease both boards.

Ethical Considerations

Anonymity and confidentiality of potential and actual participants will be ensured throughout the investigation using standard procedures in place at the WCH and the University of Toronto. All consent forms, questionnaires, data files on disks, and field notes will be stored in a locked file that will be accessed only by the investigators. No identifying information will be stored in the analytic datasets. Participants will be identified only by a study number.

### Privacy

#### Smartphone Transmissions

Patient data collected using the mobile app was double encrypted on the server and the phone. All patients will be informed how to password protect their phone. Designed from the ground up to ensure security and privacy, the app conforms to leading health care audit and interoperability standards including Personal Health Information Protection Act (PHIPA), Health Level Seven International (HL7), Information Technology Infrastructure Library (ITIL), and Statement on Auditing Standards 70 (SAS70). Multiple layers of encryption, including resting state Advanced Encryption Standard (AES) encryption, in transmission content encryption using unique per patient public / private key pairs, and in transmission Transport Layer Security / Secure Sockets Layer (TLS/SSL) protocol encryption were applied to maintain the highest level of patient confidentiality as possible. Modern infrastructure design leveraging distributed infrastructure as a service (IaaS) and software as a service (SaaS) cloud computing services for seamless accessibility, redundancy, and scalability were also utilized.

#### Research Study Data Storage

Access is restricted to the locked filing cabinets in the investigator’s office, no identifiers will be included on data sheets (only identification numbers will be used), and password-protected databases will be used. Data will be stored on a password-protected, double-encrypted, secure server. Any data stored on a mobile device (eg, universal serial bus [USB] key) will be encrypted as per WCH policies. All de-identified data will be stored for 5 years past publication and will be destroyed by shredding. The physicians will keep patient charts for the standard time period.

### Risks

There are no major identified risks from participation in this study. The “risks” of using a mobile phone or tablet device are (1) the timing of diagnosis of a complication since there is a 4% risk of complication following surgery [15], and participating in mobile versus in-person follow-up care may have an impact on the timing of diagnosis of complication; (2) security issues due the risk that someone could steal and possibly break into the contents of the phone; however, the mobile phones used in this study are double encrypted and password protected, and information is transmitted in accordance with Canadian’s Personal Information Protection and Electronic Documents Act; (3) the risk of injury from dropping the mobile phone on a foot or surgical wound, using the mobile phone while driving or operating machinery, distraction during other tasks, mobile phone overuse syndrome, and repetitive strain injury; however, the time required for the response is approximately 5 minutes; and (4) anxiety arising through possible complications from surgery, or possible loss of or damage to the device.

### Perceived Undue Influence

Patients will be generally informed about the study by their surgeon (JS or MB). The study coordinator and researcher (KA) will be responsible for the formal study explanation and informed consent process. These two people are in no way involved in the direct care of patients. All patients will be informed that their care will be in no way compromised if they choose not to participate. All patients will be informed of their ability to leave the study at any time.

### Recruitment Procedures

All patients presenting for breast reconstruction will be screened for inclusion in the study. We will consecutively approach patients to participate in the study. A member of the health care team will inform patients about the study prior to surgery. If interested in participating, a study coordinator or researcher (KA) will introduce the patient to the technology so that they can better judge what is required of them. This will include introduction to the QoR-9 and VAS score on the mobile app (see Multimedia Appendix 2). The patients will be assured of their ongoing surgical care regardless of their participation. Patients who agree to participate will review and sign the appropriate consent form. They will have the opportunity to discuss the study with the attending surgeon. Written consent will be obtained for all prospective patients. After the patient has consented to the trial, they will be randomized to the mobile app or in-person follow-up arm using the program ralloc in Stata statistical software [20].

The mobile app follow-up care stream will receive an instruction sheet detailing how to use the app. The patient will receive training on how to use the app from the study coordinator or researcher (KA). The patient will be given a mobile phone on the day of surgery and will review the use of the mobile app again at this time. This will also help the patient judge what is required of them and serve as an opt-out point.

### Data Collection Overview

**Figure 1:**
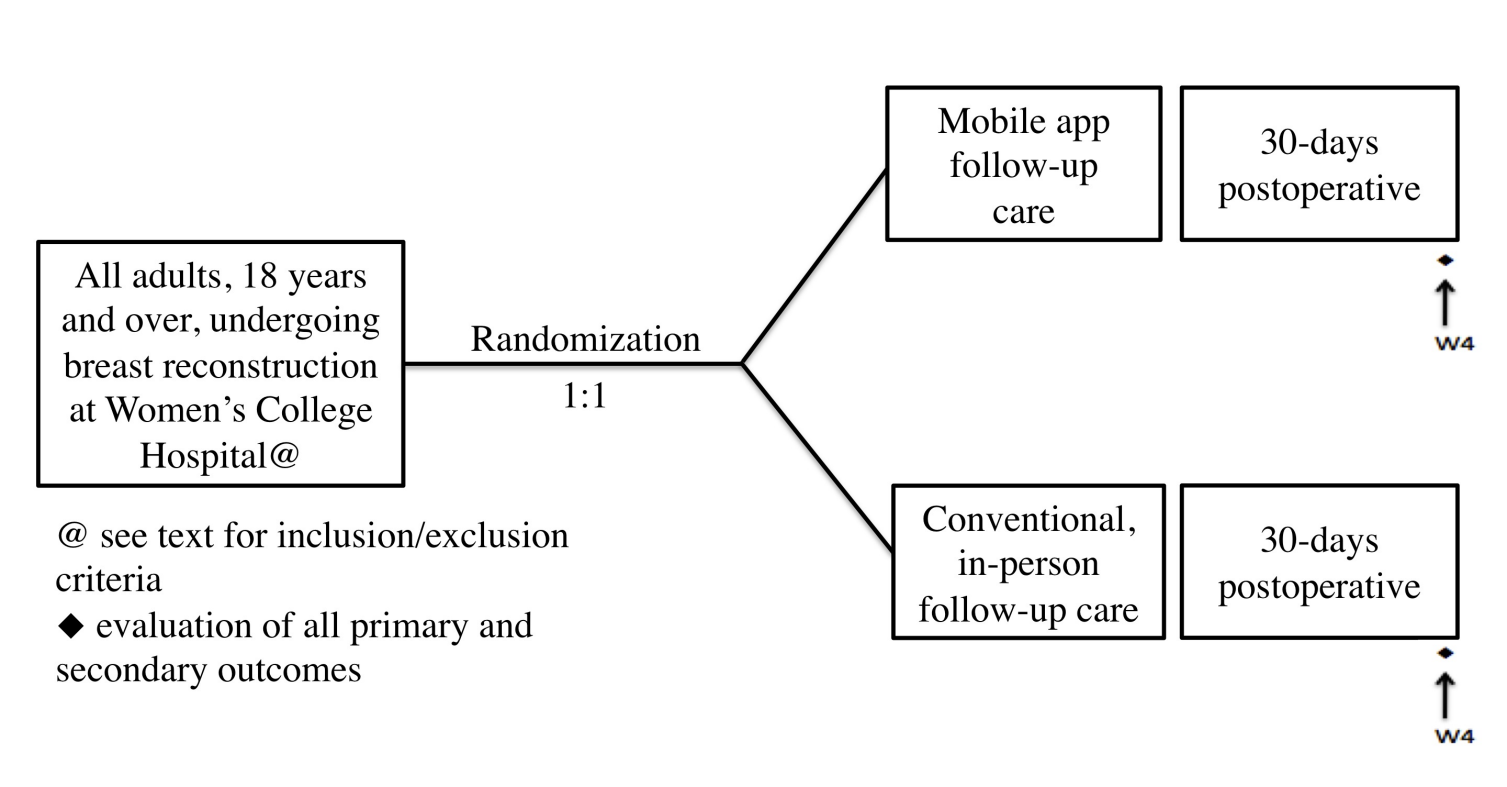
Data collection overview diagram.

The QoR and VAS pain scores will be collected from all patients at 4 weeks after surgery (see Figure 1). The in-person study group will perform this via telephone interview, and the mobile app group will perform this via mobile app. Our study coordination or researchers (KA) will collect all telephone questionnaires at 4 weeks after surgery. These persons will administer the questionnaire capturing email, telephone and in-person encounters, postoperative complications (Multimedia Appendix 3), and the cost survey (Multimedia Appendix 4). They will also administer the post-pilot survey capturing patient satisfaction (Multimedia Appendix 1) at 4 weeks after surgery. The study coordinator or researcher (KA) will attempt to contact every patient four times over the 4^th^ week after surgery.

### Measuring Outcome Variables

#### Primary Outcome

The total number of physician visits (including specialist, family physician, and emergency department) related to the surgery will be captured at 4 weeks after surgery by the study coordinator over telephone (see Multimedia Appendix 3).

#### Secondary Outcomes

The total number of health care telephone calls and emails (including specialist, family physician, and emergency department) related to the surgery will be captured at 4 weeks after surgery by the study coordinator over telephone (see Multimedia Appendix 3).

All complications will be recorded including infection, seroma, hematoma, and wound dehiscence. Among patients who receive tissue expanders, deflation is another possible complication. These complications will be collected via telephone survey at 4 weeks postoperative (see Multimedia Appendix 3).

Both mobile app and conventional follow-up patients at 4 weeks postoperative will complete our cost survey (Multimedia Appendix 4). The survey will collect data necessary to conduct cost estimates (presence of caregiver at time of follow-up, overnight hotel stays prior to clinic visit, etc).

For the purposes of this study, patients will receive a managed (loaner) mobile phone; however, a bring-your-own-device model is a more scalable solution and will be represented in the cost-effectiveness data [25].

In-person follow-up costs include foregone leisure time or income for the patient and foregone income for the caregiver, travel, and overnight hotel stay and parking costs associated with follow-up visits. Our cost survey (Multimedia Appendix 4) will collect information on caregiver presence at appointments, overnight hotel stays prior to appointments, total time commitment of in-person follow-up, and return to work information. In the literature, leisure time is often valued more than foregone income because subjects are choosing to forego their income for that leisure time. We will equally weight foregone income and leisure time, as patients are not choosing to be off work. They are off work because of a medical condition. We will determine foregone income based on average Ontario wages for a given age and sex of the patient. We will determine travel costs based on distance from home postal code to WCH.

Mobile phone follow-up costs include the foregone leisure time to submit follow-up data and the cost of data submission. We will determine foregone leisure time based on questionnaire time stamps from software records. Patient training sessions are held while patients are waiting for their preoperative appointment. There are no additional patient costs associated with this time.

In-person follow-up costs related to the health care system include the overhead (lighting, heating), physician fee, staffing costs including registrars, administrative assistance, nursing, and housekeeping. Two registrars service 32 clinic rooms. One administrative assistant and nurse is assigned to each breast reconstruction follow-up clinic. A follow-up clinic generally runs for 5 hours and serves 20 patients. WCH will provide hourly overhead and staffing costs, and OHIP billing codes will provide physician fees.

Mobile phone follow-up costs related to the health care system include the start-up costs of establishing assessment questionnaires for the patient populations to be monitored. This cost is based on the number of assessment questionnaires that need to be designed based on the diversity of the surgical population (eg, orthopedic versus general surgery patient monitoring). The QoC Health Inc mobile app can be loaded onto Android or iOS (Apple) smartphone or tablet. Formal training costs include the salary of the instructor from QoC Health Inc and the hourly salary of 1 administrative assistant, 1 postoperative ambulatory care unit nurse, and 2 physicians being trained. All staff turnover is >5 years; therefore, these costs will be amortized over 5 years. Current e-assessment OHIP physician billing codes are limited to dermatology and ophthalmology. There is no OHIP billing code for the postoperative or surgical e-assessment. The cost of software for the physician and patient and associated technical support is $3.50 (CAD) per patient per day [7]. There is discounting for hospitals based on how many patients are enrolled. Costs associated with patient training sessions include the administrative assistants’ hourly salary. The data required to send the smartphone assessment is equivalent to one email transmission.

Externally borne in-person follow-up costs include lost labor force once an individual has returned to work. In the cost survey (Multimedia Appendix 4), we will ask patients when they returned to work. We will add lost labor force costs to the in-person follow-up visits among patients who had returned to work using average Ontario wages based on the age and sex of the patient.

Patient satisfaction will be measured at 4 weeks after surgery using the post-pilot survey (Multimedia Appendix 1). The study coordinator will administer this over the telephone. The QoR-9 scores will be recorded at 4 weeks after surgery using the mobile app at home or a tablet during a scheduled clinic visit, depending on the patient’s study arm.

### Measuring Predictor Variables

Age impacts foregone wage estimates, and postal code impacts travel cost estimates. The type of surgical procedure, ASA classification, BMI, and preoperative radiation impacts rates of successful surgery at 30 days postoperative. Therefore, these variables are considered potential predictors of cost-effectiveness.

### Timeline

Any given patient may be recruited for the study up to 2 months prior to their surgery date. They will participate in the study for a total of 1 month following their surgery. Set-up and REB approval (1-2 months); a concurrent enrollment and data collection period to attain the required study participants (4-5 months); merging, cleaning, and debugging (1 month); and development of value messages for decision-makers and knowledge dissemination activities (2 months). This study will require approximately 1 year for completion. Its precise duration depends on the patient enrollment rate.

### Intervention Assignment Procedures

#### Randomization Strategy

All enrolled participants will be randomized to receive either the mobile app or conventional, in-person follow-up care in a ratio of 1:1 after giving informed consent for the study.

#### Sequence Generation

A block randomization scheme with variable block size will be generated using Stata ralloc [20]. This will ensure approximately equal sample size and that participants and study staff cannot anticipate assignment to either group. Treatments will be allocated in a 1:1 ratio. All study investigators and staff will be blinded to the block number, block size, and sequence in the block. The treatments will be assigned via pre-prepared sealed, opaque envelopes, and the envelopes will be ordered in the sequence of treatment assignments generated by the Stata code. Once eligibility for randomization has been determined, the first available allocation envelope will be assigned to the study subject. The subject will be randomized to the treatment arm indicated inside the envelope.

####  Allocation Concealment

As potential subjects are identified, a research assistant will be notified to assess eligibility. Once a subject is deemed eligible for enrollment, has given the necessary informed consent, has been enrolled, and has had her demographic information abstracted from her clinic records, the research assistant will issue the next in a series of sequentially numbered, sealed, opaque envelopes containing group assignment. The participant’s clinic number will be written on the envelope, and the participant will be required to open and read the treatment assignment in the presence of the research assistant, who will then enter the group assignment into a database and store the envelope. If a participant is unable to read, the research assistant will read the group assignment on her behalf.

####  Implementation of Randomization Procedures

A biostatistician (BZ) assigned to the study from the Institute of Health Policy, Management and Evaluation will carry out randomization procedures. The biostatistician will not be involved in any other aspects of conducting the study. The sequentially numbered, opaque, sealed envelopes containing group assignment will be given to the study coordinator. The study biostatistician will retain the key to treatment assignments. The study coordinator will be on site every day to ensure compliance with the study protocol.

## Results

### Data Analysis Plan

Descriptive statistics (frequencies, means, standard deviations) will be calculated for all clinical and outcome variables. All data obtained in this study will be entered into Excel and analyzed using Stata 13.

### Poisson Regression

We will use person-level Poisson regression to determine if there is a difference in the number of visits attended between patients in the mobile app and in-person follow-up arm.

### Net Benefit Regression

We will perform a person-level net benefit regression to determine the cost-effectiveness of this intervention. We will define cost as all societal costs incurred over the 30 days after surgery. We will define effect as the rate of complication over the 30 days after surgery. We will regress net benefit (dependent variable) on study arm, age, distance from home to hospital (km), BMI, BMI^2^, ASA classification, radiation status, and major or minor procedure (independent variables). Net benefit, age, distance, BMI, and BMI^2^ are continuous variables. ASA classification is a categorical variable. Study arm, radiation status, and major or minor procedure are binary variables (1=mobile app; 0=in-person and 1=yes, 0=no), where:

nb = B0 + B1(TX) + B2(age) + B3(km) + B4(BMI) + B5(BMI^2^) + B6(ASA) + B7(radiation) + B8(major/minor)

We will perform regression diagnostics and use these diagnostics to advise on the use of parametric versus non-parametric generation of 95% confidence intervals. We will use our net benefit regression to generate an incremental net benefit (INB):

INB = WTP * (effect_i_ – effect_c_) – (cost_i_ – cost_c_), where:

effect_i_ = mean effect of the intervention, ie, rate of complication in the mobile app arm at 30 days

effect_c_ = mean effect of the control, ie, rate of complication in the in-person arm at 30 days

cost_i_ = mean cost of the intervention, ie, societal costs from baseline to 30 days

cost_c_ = mean cost of the control, ie, societal costs from baseline to 30 days

In this situation, where willingness to pay is unknown, we assigned numerous values for willingness to pay and generated a cost-effectiveness acceptability curve (CEAC) based on these theoretical values [26]. The CEAC illustrates the probability that the intervention is cost-effective by graphing the probability that B1>0 as a function of willingness to pay (WTP) [27].

### Handling Missing Data

We will generate “best” and “worst” case scenarios for the missing data to determine if there is any change in findings.

## Discussion

### Relevance

At Women’s College Hospital, over 5000 elective ambulatory surgeries are performed each year. These numbers are small compared to other hospitals, such as the nearby Trillium Health Center, where over 20,000 surgeries are performed annually. These numbers will continue to grow as we follow trends in the United States where currently 60-70% of the surgical procedures are performed in the ambulatory setting [28]. Smartphones are becoming ubiquitous throughout Ontario. Using such an ubiquitous technological platform to reduce health care costs for patients and providers in an already large and growing patient population is in concordance with the Ontario Action Plan. Telemedicine is identified as a way to improve access to specialty care among underserviced communities [29]. Breast reconstruction is an underused option for patients, despite its well-known psychosocial benefits in breast cancer patients [2,8]. In fact, Canada underperforms next to countries like England and the United States. Particularly, low income and nonurban residence is associated with decreased rates of breast reconstruction [9]. By reducing costs to society, of which a component represents costs borne by patients, and eliminating the barriers of distance, telemedicine can improve access to breast reconstruction among breast cancer survivors.

Telemedicine literature is often criticized for its lack of rigorous economic evaluation [28]. In assessing the cost-effectiveness of mobile phone follow-up, we will determine general parameters that are necessary to make a telemedicine postoperative follow-up program more cost-effective. Upcoming telemedicine pilot programs can model costs around these general parameters.

### Knowledge Translation Plan

The study findings will yield information relevant to clinicians, hospital administrators, and decision makers regarding support and investment in telemedicine follow-up technology. If the mobile phone app (QoC Health Inc, Toronto) is demonstrated to be cost-effective, this would support its dissemination among other divisions of ambulatory surgery, including orthopedic and general surgery. It will advise policy decision makers and managers at the regional and local level regarding who are responsible for feasibility assessment, resource allocation, program design, and quality improvement. Several knowledge dissemination activities are planned. Dissemination activities will be pursued initially in the Greater Toronto Area, and eventually, nationally to ensure that the research findings reach a broad audience. To enhance knowledge dissemination, a user-friendly report will be produced using the standard 1:3:25 format recommended by the Canadian Health Services Research Foundation (1 page of key messages for decision makers; a 3-page executive summary; and a 25-page final report written in an accessible language).

### Knowledge Translation Among Health Care Providers

Locally, we will submit our findings for presentation at Gallie Day at the University of Toronto. Gallie Day attracts surgeons from all fields at the University of Toronto. Surgeons who attend this event tend to have a hand in either research or hospital administration. They work at various hospitals throughout the Greater Toronto Area. And we will present our findings at the American Society of Plastic Surgery Annual Meeting. This meeting tends to draw as many Canadians as the Canadian Society of Plastic Surgery Annual Meeting, as well as American and international plastic surgeons. This is a suitable population for dissemination as most plastic surgeons perform a large quantity of elective ambulatory surgeries. We will aim for publication in *Canadian Medical Association Journal* or *JAMA Surgery*. These journals attract a breadth of readers that include plastic surgeons and other types of ambulatory surgeons.

### Knowledge Translation Among Administrators and Policy Decision Makers

We will submit our findings for presentation at the Ontario Hospital Association Conference and Health Achieve conference. These conferences tend to attract administrators and policy decision makers. Findings will be further communicated nationally through use of various listservs.

To facilitate the research process and to enhance involvement, 2 or more members of the team meet weekly. Such meetings foster communication and progress. The clinicians (JS and MB) on our team are enthusiastic to improve ease of follow-up with telemedicine. They have indicated that they are ready to champion research results within their respective organizations by narrowing the gap between evidence and action, and they wish to be proponents of evidence-informed decision making for the system and policy environment.

### Limitations

There are three main study limitations. First, the timeline of data collection is limited to the first 30 days postoperatively. This reference was based on literature around the first 30 days postoperative. Studies reveal that the 2-week period after hospital discharge is the most vulnerable time and 75% of complications show up within 14 days of discharge [21]. Complications (eg, capsular contracture) beyond 30 days represent the minority; we will minimize bias by using the same timeline of data collection in both the telemedicine and conventional follow-up group.

A second limitation lies in the potential generalizability of the findings. Study participants will be drawn from those receiving ambulatory breast reconstruction at WCH, and consequently, the findings may not necessarily generalize to patients receiving in-patient breast reconstruction (eg, bilateral deep inferior epigastric perforator [DIEP] flap). However, the populations served are quite diverse in terms of their clinical, demographic, and regional background, which may help to improve generalizability to elective ambulatory surgery patients including the majority of breast reconstruction performed in the community. In addition, because only female English-speaking participants without psychiatric condition or chronic pain syndrome will be recruited, the findings may not be generalizable to certain populations (eg, non-English speaking).

A third limitation surrounds self-reporting and estimating costs. We will rely on in-person follow-up patients to provide us with estimated time of travel from home to clinic and back to home. We will limit bias by correlating this with distance from clinic information based on postal code. We also must rely on average wage estimates from Statistics Canada and average travel costs from Canadian Automobile Association. These estimates are commonly used in cost-effectiveness literature.

### Conclusion

This study will determine if mobile app technology can be used to replace in-person follow-up care after ambulatory surgery. Such replacement may have multiple secondary ramifications including cost-effectiveness and patient satisfaction.

## Appendix

Multimedia Appendix 1 Post-pilot satisfaction survey capturing patient satisfaction with care and information provided for all patients, to be completed by all patients at week four.

Multimedia Appendix 2 QoR9 and Pain VAS.

Multimedia Appendix 3 Telephone questionnaire capturing email, telephone and in-person encounters and postoperative complications. All patients will complete this at week four.

Multimedia Appendix 4 Telephone questionnaire capturing patient costs, to be completed by all patients at week two and week four.

Multimedia Appendix 5 CIHR reviewer comments.

## Abbreviations

ASA: American Society for Anesthesiologists
BMI: body mass index
CEAC: cost-effectiveness acceptability curve
INB: incremental net benefit
OHIP: Ontario Health Insurance Plan
QoR: quality of recovery
QoR-9: Quality of Recovery-9
QoR-40: Quality of Recovery-40
REB: Research Ethics Board
VAS: visual analog scale
WCH: Women’s College Hospital
WTP: willingness-to-pay
