# Supplementary material for: The Effect of Mobile App Home Monitoring on Number of In-Person Visits Following Ambulatory Surgery: Protocol for a Randomized Controlled Trial
Source: JMIR Res Protoc. 2015 Jun 3;4(2):e65. doi: 10.2196/resprot.4352 (PMC4526905; doi:10.2196/resprot.4352)
Supplement: Multimedia Appendix 1 [file resprot_v4i2e65_app1.pdf]

WOMEN'S COLLEGE HOSPITAL
Health care for women | REVOLUTIONIZED

Replacing Ambulatory Clinic Follow-up with Mobile App Home Monitoring in Breast Reconstruction Patients: A Randomized Controlled Trial

Post-Pilot Survey capturing patient satisfaction with care and information provided for all patients, to be completed by all patients at week four.

Post-Pilot
Satisfaction Survey

Patient Identification No: ________________________ **Date:** ________________________

We would like to learn more about the quality of your recovery at home. Please take a few minutes to fill in this questionnaire. Your responses are confidential and will be grouped with others responses to ensure anonymity. Your participation in filling in the survey is voluntary and will not affect your care.

The following questions will help us to understand your satisfaction with care.

1. My overall satisfaction with the care after surgery was
□ excellent □ good □ fair □ poor

2. Were you satisfied with the type of follow-up care (mobile app or in-person) you received?
□ strongly agree □ agree □ neutral □ disagree □ strongly disagree □ n/a

3. Would you describe the type of follow-up care (mobile app or in-person) you received as convenient?
□ strongly agree □ agree □ neutral □ disagree □ strongly disagree □ n/a

4. Availability of doctors: how easy it was to get in contact with your doctor(s) when needed
□ excellent □ very good □ good □ fair □ poor □ n/a

5. I was sent home from the hospital before I felt ready
□ strongly agree □ agree □ neutral □ disagree □ strongly disagree □ n/a

The following questions will help us to understand your satisfaction with information you received.

6. I knew who to contact if I had problems following discharge

□ strongly agree □ agree □ neutral □ disagree □ strongly disagree □ n/a

7. I was told what to expect when I got home
□ strongly agree □ agree □ neutral □ disagree □ strongly disagree □ n/a

8. I learned what I needed to know in order to manage my condition at home
□ strongly agree □ agree □ neutral □ disagree □ strongly disagree □ n/a

9. My family/friends received information to assist in providing care for me at home
□ strongly agree □ agree □ neutral □ disagree □ strongly disagree □ n/a

10. Before leaving the hospital, I was given adequate information about how to monitor my condition for problems and danger signals
□ strongly agree □ agree □ neutral □ disagree □ strongly disagree □ n/a

11. My emotional needs (worries, fears, anxieties) were acknowledged and addressed
□ strongly agree □ agree □ neutral □ disagree □ strongly disagree □ n/a

Thank you for filling out our survey, your feedback is very appreciated!
