# Supplementary material for: The Effect of Mobile App Home Monitoring on Number of In-Person Visits Following Ambulatory Surgery: Protocol for a Randomized Controlled Trial
Source: JMIR Res Protoc. 2015 Jun 3;4(2):e65. doi: 10.2196/resprot.4352 (PMC4526905; doi:10.2196/resprot.4352)
Supplement: Multimedia Appendix 2 [file resprot_v4i2e65_app2.pdf]

WCH
WOMEN'S COLLEGE HOSPITAL
Health care for women | REVOLUTIONIZED

Replacing Ambulatory Clinic Follow-up with Mobile App Home Monitoring in Breast Reconstruction Patients: A Randomized Controlled Trial

Questionnaires to be completed by telemedicine follow-up care patients daily for the first two weeks, and weekly for the subsequent 2 weeks; and by the conventional follow-up care patients during their scheduled clinic visit at one and four weeks postoperative.

Patient Identification No: _______________ Date: ____________________

1. How anxious (worried, nervous) do you feel?
   - not at all anxious
   - a little anxious
   - moderately anxious
   - very anxious
   - extremely anxious

2. Please indicate the level of pain you are feeling right now.

[Visual Analogue Scale]

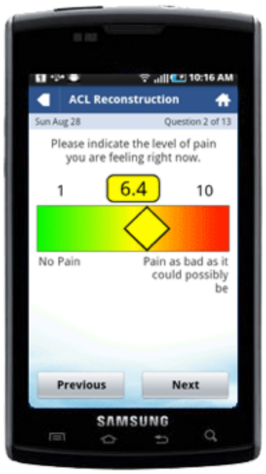

The following QOR-9 will be ranked on a 5-point Likert scale:
1-none of the time, 2-some of the time, 3-usually, 4-most of the time, 5-all of the time

3. Had a feeling of general well being

4. Had support from others

5. Been able to understand instructions and advice. Not being confused

6. Been able to look after personal toilet and hygiene unaided

7. Been able to pass urine ("waterworks") and having not trouble with bowel function

8. Been able to breathe easily

9. Been free from headache, backache or muscle pains

10. Been free from nausea, dry-retching or vomiting
11. Been free from experiencing severe pain or constant moderate pain

12. Feeling comfortable and in control

13. Amount of fluid drained from wound (in cc's): _________________
