# Supplementary material for: The Effect of Mobile App Home Monitoring on Number of In-Person Visits Following Ambulatory Surgery: Protocol for a Randomized Controlled Trial
Source: JMIR Res Protoc. 2015 Jun 3;4(2):e65. doi: 10.2196/resprot.4352 (PMC4526905; doi:10.2196/resprot.4352)
Supplement: Multimedia Appendix 3 [file resprot_v4i2e65_app3.pdf]

WOMEN'S COLLEGE HOSPITAL
Health care for women | REVOLUTIONIZED

Replacing Ambulatory Clinic Follow-up with Mobile App Home Monitoring in Breast Reconstruction Patients: A Randomized Controlled Trial

Telephone questionnaire capturing email, telephone and in-person encounters and postoperative complications. All patients will complete this at week two and week four.

Patient Identification No: ______________________ **Date:** ______________________

1. Did you want to be in contact with your health care provider/surgeon but <u>did not</u> call/email/visit?

□ yes
□ no

If "yes", please describe what kind of contact you wanted.

____

____

2. Did you <u>call</u> the hospital/your surgeon/your nurse while you were recovering at home, before your first or second follow-up appointment?

□ yes, before my □ first appointment and/or □ second appointment
□ no

If "yes", please describe why

____

____

3. Did you <u>email</u> the hospital/your surgeon/your nurse while you were recovering at home, before your first or second follow-up appointment?

□ yes
□ no

If "yes", please describe why

____

____

4. Did you visit the emergency department/hospital/your surgeon/your nurse (unscheduled) while you were recovering at home, during the first month after surgery?
□ yes
□ no

If "yes", please describe why
____

____

5. (Seroma) Did you experience a collection of fluid under the skin that required drainage by needle?
□ yes
□ no

6. (Hematoma) Did you experience a collection of blood under the skin that required drainage by needle or another stay in the hospital or surgery?
□ yes
□ no

7. (Infection) Did you experience redness and/or pus around a site of operation that required an antibiotic prescription?
□ yes
□ no

8. (Wound dehiscence) Did you experience a wound that required prolonged nursing care or another procedure the clinic or in the operating room?
□ yes
□ no

9. Please describe any complications that you experienced related to your breast surgery:
____

____
