# Supplementary material for: The Effect of Mobile App Home Monitoring on Number of In-Person Visits Following Ambulatory Surgery: Protocol for a Randomized Controlled Trial
Source: JMIR Res Protoc. 2015 Jun 3;4(2):e65. doi: 10.2196/resprot.4352 (PMC4526905; doi:10.2196/resprot.4352)
Supplement: Multimedia Appendix 4 [file resprot_v4i2e65_app4.pdf]

WOMEN'S COLLEGE HOSPITAL
Health care for women | REVOLUTIONIZED

Replacing Ambulatory Clinic Follow-up with Mobile App Home Monitoring in Breast Reconstruction Patients: A Randomized Controlled Trial

Telephone questionnaire capturing patient costs, to be completed by all patients at week two and week four.

Patient Identification No: ______________________ Date: ______________________

We would like to learn more about the patient borne costs for visiting a doctor. Your participation in filling in the survey is voluntary and will not affect your care.

---

1. Did you attend a scheduled, in-person follow-up appointment with your surgeon?
□ yes
□ no

Only if yes… please complete the rest of the survey

2. Are you currently employed or seeking employment?
□ yes
□ no

If yes…
3. When did you go back to work: ____________(days after your surgery)

4. Did you stay in a hotel prior to your postoperative follow-up appointment?
□ yes
□ no
□ N/A (telemedicine follow-up)

5. How much time does it take to attend a follow-up appointment (from time you leave your house, until time you arrive back at home)? __________ minutes __________ hours

6. Did a caregiver (e.g. partner, child, friend) attend the postoperative clinic visits with you?
□ yes
□ no
□ N/A (telemedicine follow-up)

If yes…
7. Is your caregiver currently employed or seeking employment?

□ yes
□ no

8. Please provide the birth year _________, sex _________ of the caregiver attending the visit with you. (Estimates within 5 years are acceptable.)

Thank you for your participation in this study!
