# Supplementary material for: The Effect of Mobile App Home Monitoring on Number of In-Person Visits Following Ambulatory Surgery: Protocol for a Randomized Controlled Trial
Source: JMIR Res Protoc. 2015 Jun 3;4(2):e65. doi: 10.2196/resprot.4352 (PMC4526905; doi:10.2196/resprot.4352)
Supplement: Multimedia Appendix 5 [file resprot_v4i2e65_app5.pdf]

Review Type/Type d'évaluation: Committee Member 1/Membre de comité 1
Name of Applicant/Nom du chercheur: SEMPLE, John Laurie
Application No./Numéro de demande: 316807
Agency/Agence: CIHR/IRSC
Competition/Concours: 2013-10-15 Catalyst Grant: e-Health Innovations: Supporting More Efficient Population and Individualized Healthcare/Subvention catalyseur : Innovations en cybersanté : soutenir l'amélioration des soins de santé axés sur les individus et les populations
Committee/Comité: e-Health Innovations/Innovations en cybersanté
Title/Titre: Replacing ambulatory clinic follow-up with remote home monitoring using smartphones in breast reconstruction patients: Is it cost-effective?

Assessment/Évaluation:

SEMPLE – Replacing ambulatory clinic follow-up with remote home monitoring using smartphones in breast reconstruction patients: Is it cost-effective?

A brief synopsis of the proposal

Evaluating the use of smartphones for followup of breast reconstructive surgery versus standard followup.

Strengths

This proposal strengths are the qualifications of the research team and its focus on cost-effectiveness and the needs of the health system in which this research will inform. The group has partnered with a Canadian-based SME and is intending to examine economic impact in order to create a value position and more widespread use.

Limitations

There are some minor limitations in the proposal. Mostly there is a need to make standard references in regards to methods (e.g., adherence to CADTH or ISPOR best practice guidelines) to assure reviewers this framework will be used.

Review Type/Type d'évaluation: Committee Member 2/Membre de comité 2
Name of Applicant/Nom du chercheur: SEMPLE, John Laurie
Application No./Numéro de demande: 316807
Agency/Agence: CIHR/IRSC
Competition/Concours: 2013-10-15 Catalyst Grant: e-Health Innovations: Supporting More Efficient Population and Individualized Healthcare/Subvention catalyseur : Innovations en cybersanté : soutenir l'amélioration des soins de santé axés sur les individus et les populations
Committee/Comité: e-Health Innovations/Innovations en cybersanté
Title/Titre: Replacing ambulatory clinic follow-up with remote home monitoring using smartphones in breast reconstruction patients: Is it cost-effective?

Assessment/Évaluation:

1. **Grant Title: #316807. Replacing ambulatory clinic follow-up with remote home monitoring using smartphones in breast reconstruction patients: Is it cost-effective?**

2. **Brief Synopsis of Proposal**

The proposal describes a small prospective cohort in one hospital to compare the cost-effectiveness of a mobile follow-up for breast reconstructive surgery versus usual in-person follow-up (2 clinics) at 30 days.

3. **Research Approach**

The grant is well written and easy to follow.

The authors outline well the rationale for providing mobile phone-related follow-up after surgery since patients come from all over Ontario and have large travel distances, the Ontario government is interested in technology-based efficiencies in healthcare, and the complication rate requiring intervention after this surgery is low.

A feasibility study of a case series of 30 patients showed satisfactory patient acceptance and outcomes with the mobile app QoC. Further information on QoC - why it was selected, what competitive process was followed, what are its features, etc.

The intervention app, which is expected to be loaded on the patient's own phone. The app includes pictures transmitted of the surgical site, a short quality of recovery questionnaire, visual analogue scale on pain.

Given that the study is to be prospective, it is unclear why the applicants do not randomize. This important move as well as blinded outcome assessment, would improve the study considerably.

The study is billed as a cost-effectiveness analysis yet the authors have not described a primary outcome. Given that the assumption appears to be that mobile monitoring will be as effective as conventional monitoring, but cheaper implies that a cost-minimization analysis might be appropriate. Clarification of the economic plan is needed.

Regardless of the economic plan, a primary outcome should be declared *a priori;* the definition of 'surgical success' must be declared.

**Review Type/Type d'évaluation:**	Committee Member 2/Membre de comité 2
**Name of Applicant/Nom du chercheur:**	SEMPLE, John Laurie
**Application No./Numéro de demande:**	316807
**Agency/Agence:**	CIHR/IRSC
**Competition/Concours:**	2013-10-15 Catalyst Grant: e-Health Innovations: Supporting More Efficient Population and Individualized Healthcare/Subvention catalyseur : Innovations en cybersanté : soutenir l'amélioration des soins de santé axés sur les individus et les populations
**Committee/Comité:**	e-Health Innovations/Innovations en cybersanté
**Title/Titre:**	Replacing ambulatory clinic follow-up with remote home monitoring using smartphones in breast reconstruction patients: Is it cost-effective?

Assessment/Évaluation:

More information of patient health information privacy, governance and the handling of intellectual property, would have been welcome.

I am surprised that the applicants do not mention a major barrier to telemedicine, and how they plan to deal with it – how will the physicians bill for their time if not seeing the patient in person, and what specialized equipment is required?

4. Originality
Very good clinical topic and very good to see focus on economics of technical solution

5. End-user Requirements or Preferences Incorporated?
Not evident

6. Budget
Budget is very parsimonious and justified. The use, where possible, of the patient's own phone in the study increases the generalizability of the results. However, it may well cut down on the recruitable patient group. Most older patients do not use smart phones.

7. Applicants
The team is small - includes an experienced surgeon-researcher, a senior health economist, a plastic surgery resident in a Master's program, and a physician-director in the hospital as knowledge user. Addition of a clinical trials expert would have been a plus.

8. Impact of the Research – potential to augment, integrate, evaluate e-health approaches; add value to existing service delivery; improve quality, equity, cost-effectiveness
Since research is being carried out in a busy breast centre and results will be readily disseminated and would be relatively simple to generalize, there could be considerable impact to change the paradigm of follow-up care.

9. Environment for Research
Seems very good.

**Review Type/Type d'évaluation:**	Committee Member 2/Membre de comité 2
**Name of Applicant/Nom du chercheur:**	SEMPLE, John Laurie
**Application No./Numéro de demande:**	316807
**Agency/Agence:**	CIHR/IRSC
**Competition/Concours:**	2013-10-15 Catalyst Grant: e-Health Innovations: Supporting More Efficient Population and Individualized Healthcare/Subvention catalyseur : Innovations en cybersanté : soutenir l'amélioration des soins de santé axés sur les individus et les populations
**Committee/Comité:**	e-Health Innovations/Innovations en cybersanté
**Title/Titre:**	Replacing ambulatory clinic follow-up with remote home monitoring using smartphones in breast reconstruction patients: Is it cost-effective?

Assessment/Évaluation:

10. Alignment with Funding Opportunity

Excellent

11. Innovative Partnerships with e-health Enterprises?

Not mentioned; as above, would be useful to hear more on relationships with mobile app company

Review Type/Type d'évaluation: SO Notes /Notes de l'agent scientifique
Name of Applicant/Nom du chercheur: SEMPLE, John Laurie
Application No./Numéro de demande: 316807
Agency/Agence: CIHR/IRSC
Competition/Concours: 2013-10-15 Catalyst Grant: e-Health Innovations: Supporting More Efficient Population and Individualized Healthcare/Subvention catalyseur : Innovations en cybersanté : soutenir l'amélioration des soins de santé axés sur les individus et les populations
Committee/Comité: e-Health Innovations/Innovations en cybersanté
Title/Titre: Replacing ambulatory clinic follow-up with remote home monitoring using smartphones in breast reconstruction patients: Is it cost-effective?

Assessment/Évaluation:

SEMPLE
This is a strong proposal that that deals with an application has already been developed and includes validated instruments. It is a small but well qualified team with relevant experience. The proposal did not refer to an economic evaluation framework and a primary outcome was not clearly stated. It was unclear why the study did not include randomization and blinded outcomes in place of an observation study. Data security/privacy issues were not addressed.
